# Anticancer Mechanism of Flavonoids on High-Grade Adult-Type Diffuse Gliomas

**DOI:** 10.3390/nu15040797

**Published:** 2023-02-04

**Authors:** Shu Chyi Wong, Muhamad Noor Alfarizal Kamarudin, Rakesh Naidu

**Affiliations:** Jeffrey Cheah School of Medicine and Health Sciences, Monash University Malaysia, Jalan Lagoon Selatan, Bandar Sunway 47500, Selangor Darul Ehsan, Malaysia

**Keywords:** flavonoids, anticancer, high-grade adult-type diffuse gliomas

## Abstract

High-grade adult-type diffuse gliomas are the most common and deadliest malignant adult tumors of the central nervous system. Despite the advancements in the multimodality treatment of high-grade adult-type diffuse gliomas, the five-year survival rates still remain poor. The biggest challenge in treating high-grade adult-type diffuse gliomas is the intra-tumor heterogeneity feature of the glioma tumors. Introducing dietary flavonoids to the current high-grade adult-type diffuse glioma treatment strategies is crucial to overcome this challenge, as flavonoids can target several molecular targets. This review discusses the anticancer mechanism of flavonoids (quercetin, rutin, chrysin, apigenin, naringenin, silibinin, EGCG, genistein, biochanin A and C3G) through targeting molecules associated with high-grade adult-type diffuse glioma cell proliferation, apoptosis, oxidative stress, cell cycle arrest, migration, invasion, autophagy and DNA repair. In addition, the common molecules targeted by the flavonoids such as Bax, Bcl-2, MMP-2, MMP-9, caspase-8, caspase-3, p53, p38, Erk, JNK, p38, beclin-1 and LC3B were also discussed. Moreover, the clinical relevance of flavonoid molecular targets in high-grade adult-type diffuse gliomas is discussed with comparison to small molecules inhibitors: ralimetinib, AMG232, marimastat, hydroxychloroquine and chloroquine. Despite the positive pre-clinical results, further investigations in clinical studies are warranted to substantiate the efficacy and safety of the use of flavonoids on high-grade adult-type diffuse glioma patients.

## 1. Introduction

According to the World Health Organization (WHO), adult-type diffuse gliomas account for the majority of primary adult brain tumors in adult neuro-oncology practice. High-grade adult-type diffuse gliomas are aggressive brain tumors that account for up to 85% of all new cases of malignant primary brain tumors diagnosed every year, with an incidence of approximately 5 per 100,000 persons in Europe and North America [1]. Glioblastoma (GBM) and grade 4 astrocytoma, IDH-mutant are the most common high-grade adult-type diffuse gliomas and contribute approximately 70% of the total cases [2]. The remaining 15% are grade 3 astrocytoma, IDH-mutant, and 10% are grade 3 oligodendroglioma, IDH-mutant and 1p/19q co-deleted [2]. The survival rate of adult-type diffuse glioma patients greatly depends on the subtype of glioma present, with five-year survival rates as high as 80% in low-grade glioma, while it is only under 5% in high-grade glioma patients [3]. The current standard treatment for high-grade adult-type diffuse glioma patients includes maximal surgical resection, followed by concomitant temozolomide (TMZ) and radiation, then several cycles of adjuvant alkylating drugs TMZ [4]. Other than TMZ, there are four drugs and one FDA-approved device for the treatment of high-grade adult-type diffuse gliomas: lomustine, intravenous carmustine, carmustine wafer implants, bevacizumab and tumor-treating fields (TTF, Optune) [5]. However, adverse events or dose-limiting toxicities of these alkylating drugs always cause early discontinuation of treatment [6]. Most importantly, widespread exposure to alkylating drugs and the highly heterogeneous and mutation-prone nature of high-grade adult-type diffuse gliomas can lead to chemoresistance [7,8]. Hence, introducing new therapeutic strategies that can target multiple mechanisms with low toxicity is much needed to lead to a successful high-grade adult-type diffuse glioma treatment.

Historically, naturally occurring polyphenols have played a key role in drug discovery for infectious diseases, cardiovascular disease, multiple sclerosis and, most importantly, cancer [9,10,11]. Polyphenols are the most popular secondary metabolites existing in plants, abundantly found in tea, vegetables, fruits and wine. Polyphenol structural skeletons have an aromatic ring with one or more hydroxyl groups. They are further classified according to their chemical structure, with four main subdivisions, which are phenolic acids, flavonoids, stilbenes and lignans. Among them, flavonoids comprise the most well-studied bioactive compound due to their diverse health benefits and wide distribution. There are six different categorizations under flavonoids, which are flavonols, flavones, flavanols, flavanones, isoflavones and anthocyanidins [12,13]. Numerous studies have discovered their antioxidant, antimicrobial, antiviral, anti-inflammatory and strong anticancer properties that can be used in the treatment of various diseases [12,14]. Flavonoids, as an antioxidant compound, improve vascular health and reduce the risk of cardiovascular disease in their conjugated form [15]. Flavonoids also prevent age-related neurodegenerative diseases such as dementia, Parkinson’s and Alzheimer’s disease [16]. Most importantly, flavonoids can modulate several molecular activities and induce apoptosis, cell cycle, and autophagy to suppress cancer cell proliferation and invasiveness [17].

With the ability to cross the BBB and a known ability to impact cancer processes such as proliferation, differentiation, apoptosis, and autophagy via multiple molecular targets, flavonoids have been widely studied for high-grade adult-type diffuse glioma treatment [18,19,20]. Flavonoids inhibit the migration and invasion of GBM and astrocytoma cells through the inhibition of MMP-2, MMP-9, Erk and p38 protein expression and activities [21]. Flavonoids also modulate the MAPK, p53 and NF-κB pathways and their downstream targeted proteins, such as the Bax/Bcl-2 ratio and caspase-3 and -9 in GBM and astrocytoma cells [22]. Moreover, flavonoids have demonstrated their anti-tumor effects in high-grade adult-type diffuse glioma by regulating autophagy-related proteins such as LC3, beclin-1 and p62 expression. It is worth noting that flavonoids also intensify the effect of conventional high-grade adult-type diffuse glioma therapies and prevent the DNA repair process by inhibiting PARP and MGMT activities [17,22]. Flavonoids have been reported to exert preferential cytotoxicity against cancer over non-cancer cell lines [23,24]. In such cases, flavonoids have become potential anti-cancer agents for high-grade adult-type diffuse gliomas, which might provide higher therapeutic effects and lower toxicity [25].

To date, numerous pre-clinical studies have suggested flavonoids as potential anti-cancer agents for high-grade adult-type diffuse gliomas [26,27,28,29]. However, a greater focus on flavonoids’ anti-high-grade adult-type diffuse glioma potential in targeting different molecules is needed to provide a more comprehensive understanding of its therapeutic effects. This review includes the initial to recent pre-clinical studies of flavonoids’ mechanism of action in modulating molecular targets involved in high-grade adult-type diffuse glioma cell proliferation, apoptosis, oxidative stress, cell cycle arrest, migration, invasion, autophagy and DNA repair [30,31,32,33,34,35,36,37,38]. Specific flavonoid compounds from each subclass were chosen based on their popularity of being studied in pre-clinical studies. This review paper also identifies the common molecular targets of different subclasses of flavonoids against high-grade adult-type diffuse glioma. Interactions between the common molecular targets are analyzed and grouped into clusters using online bioinformatics software (STRING). Additionally, the clinical relevance of the common molecular targets regulated by these flavonoids is discussed compared to the small molecule inhibitors.

## 2. Methodology

A literature search was performed across three electronic databases (PubMed, Scopus, and Google Scholar), where only English articles published up until August 2022 were included. The following search terms and Boolean connectors were involved in the search strategy: “Glioblastoma OR high-grade glioma OR adult-type diffuse glioma OR grade 4 astrocytoma OR grade 3 oligodendroglioma OR grade 3 astrocytoma” AND “flavonoid OR flavonoids OR flavonol OR flavonols OR flavone OR flavones OR flavanone OR flavanones OR flavanol OR flavanols OR isoflavone OR isoflavones OR anthocyanin OR anthocyanins”. These terms were searched in the abstract, title or keywords. From the searched results, an example of compounds in each flavonoid subclass that had been studied the most was selected. The inclusion criteria for the articles were the use of flavonoids on high-grade adult-type diffuse glioma cell lines with protein or mechanism analysis carried out in vivo or in vitro. Studies without protein or mechanism analysis were excluded. STRING analysis was used to predict the interactions between the proteins by analyzing the network edges of evidence, confidence, and molecular actions between the proteins. The interactions between the proteins were probed with the minimum required interactions score set at a high confidence level of 0.7000. The clustering of the proteins was also done using the database K-means clustering by specifying the number of clusters to three.

## 3. Flavonoids and the Mechanism of Action against High-Grade Adult-Type Diffuse Gliomas

**Figure 1:**
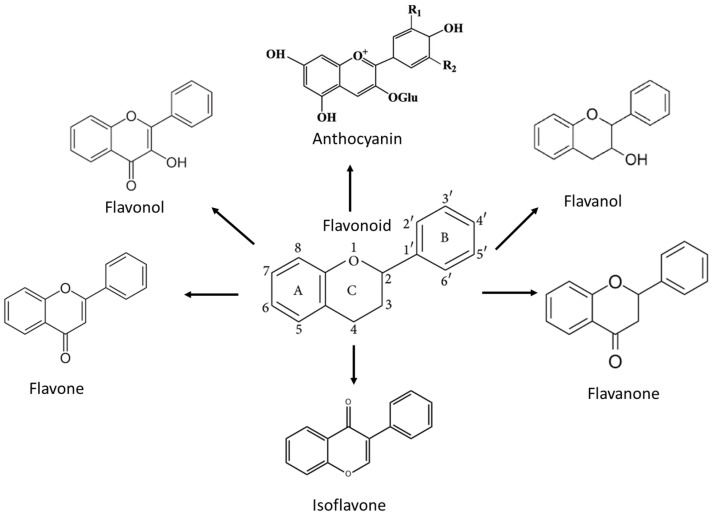
Basic chemical structure of flavonoids and their subclasses.

Flavonoids belong to a class of low-molecular-weight phenolic compounds synthesized in plants which are responsible for their color, flavor and pharmacological activities [12]. They are most commonly found in fruits, herbs, stems, flowers, vegetables and seeds. The presence of bioactive phytochemical constituents present in different parts of plants provides them with a variety of medicinal values and biological activities [39]. Thus far, scientist have discovered more than 10,000 flavonoids, and the list of newly discovered flavonoids continues to grow [39,40,41]. Flavonoids are comprised of two phenyl rings (A and B) linked via a heterocyclic pyrene ring (C ring), as shown in Figure 1. They can be further divided into a variety of classes: flavonols, flavones, flavanols, flavanones, isoflavones and anthocyanidins. This categorization depends on the degree of oxidation and pattern of substitution of the C ring, while individual compounds within a class differ in the pattern of substitution of the A and B rings, as shown in Table 1 [42]. Studies demonstrated that the position and number of substituents in the flavonoid basic structure significantly affect their biological functions in treating different diseases [43,44,45].

### 3.1. Flavonol

Flavonols are flavonoids with a ketone group. Flavonol’s structure exhibits a double bond between carbons 2 and 3 and a hydroxyl group in carbon 3 of the C ring. Examples of flavonols are kaempferol, quercetin, myricetin and rutin. Among the subclasses of flavonols, quercetin and rutin are the most well-studied compounds against high-grade adult-type diffuse gliomas.

#### 3.1.1. Quercetin

Quercetin is largely found in *quercus* leaves, apples, berries and most parts of these plants [46,56]. Quercetin is also found in medicinal botanicals, such as *Ginkgo biloba*, *Hypericum perforatum* and *Sambucus canadensis* [57]. Due to reasonable doses of quercetin having no obvious adverse side effects on normal cells, more and more research has been focused on its therapeutic effect on brain tumors. In one of the pre-clinical studies, quercetin has been shown to be selective towards high-grade adult-type diffuse glioma cells by sparing 85% of normal astrocytes alive [23]. Numerous studies have also shown that quercetin exerts anti-tumor effects in a variety of mechanisms, with encouraging results. Quercetin significantly prevents high-grade adult-type diffuse glioma tumor growth.

Studies have shown that quercetin can inhibit U87 cell growth by suppressing the phosphorylation of Akt and Erk protein levels after 48 h of treatment [58]. In the same study, quercetin downregulated MMP-9, thus inhibiting cell migration and invasion [58,59,60]. Other than that, quercetin can activate JNK, which then inhibits U373MG cell proliferation and differentiation and subsequently induces apoptosis [30]. Activation of JNK has been shown to directly or indirectly modulate the expression levels of p53 and its target genes [61]. Other than JNK, Erk and Akt protein, quercetin also inhibited the phosphorylation of p38 and RAF-1 and MYC reporters [62,63]. The suppression of Akt levels is stronger at quercetin post-treatment compared to the combination of temozolomide plus irradiation [63]. The treatment of human U251 and TG1 cells with quercetin and rutin also suppresses the expression of mRNA for IL-6 and IL-10, which inhibit proliferation and migration rate [64].

Quercetin can decrease the expression of autophagy proteins such as beclin-1 and LC3BII to inhibit protective autophagy and enhance apoptosis in T98G cells [30,65]. A combination of quercetin and sodium butyrate can synergistically reduce the expression of beclin-1 and LC3B-II. Other than that, quercetin suppresses Atg7 expression, which then blocks the autophagy process in U251 and U87 cells [57]. Atg7 functions as an enzyme necessary for Atg12-Atg5 conjugation and regulates the transformation of LC3 from LC3-I to LC3-II, which is essential for autophagosome formation. Quercetin also blocked autophagy activities induced by a selective soluble epoxide hydrolase inhibitor, trans-AUCB (t-AUCB), and eliminated the resistance to t-AUCB in T98G cells. Additionally, the combination of quercetin and sorafenib decreases the expression of beclin-1 and the conversion of LC3-I into LC3-II in T98G cells [66]. Other than the combination with sorafenib, quercetin and t-AUCB has been shown to sensitize T98G cells to TMZ and imperatorin by inhibiting Hsp27 and increasing caspase-3 activity [26,67,68].

Quercetin alone can cause PARP degradation in T98G, U373 and rat C6 cells [30,65]. The degradation of PARP drastically increased caspase-3 activation, leading to U373MG cell apoptosis. In the same study conducted by Kim et al., quercetin increased the expression of p53, which translocated to the mitochondria and simultaneously led to the release of cytochrome C from the mitochondria to the cytosol [30]. Other than p53, quercetin also increased caspase-3 and -9 activities [35,69]. Quercetin also reduced the recruitment of STAT3 at the cyclin D1 promoter and inhibited Rb phosphorylation in the presence of IL-6, subsequently inducing U87MG, U373MG and T98G cell apoptosis [23,70].

#### 3.1.2. Rutin

Rutin is abundantly found in ruta graveolens plants and other plants such as passionflower, onion, mulberry, buckwheat, tea and apple [47,71,72]. Rutin is also called rutoside or quercetin-3-rutinoside; chemically, it is a glycoside comprising flavonolic aglycone quercetin along with disaccharide rutinose. It has demonstrated several pharmacological activities, including antioxidant, cytoprotective, cardioprotective and anticarcinogenic properties [73].

Rutin was shown to enhance TMZ cytotoxicity in U87-MG, D54-MG, and U251-MG cell lines by inhibiting JNK phosphorylation, which is augmented by TMZ. Rutin can also downregulate Erk activation, thereby inducing death in U251 and DBTRG-05MG cell lines [74]. Erk1/2 phosphorylation in GL-15 cells was also markedly decreased with 10–100 μM rutin [75]. In a study conducted by Pingde Zhang et al., they found that TMZ treatment in the human GBM cell lines D54-MG, U87-MG and U251-MG induced cytoprotective autophagy, which compromises its treatment efficacy. Rutin, on the other hand, suppressed LC3II expression, which then increased the treatment effect of TMZ [31]. Furthermore, rutin induced U-118 and LN-229 cell death through p53 up-regulation and caused a loss of mitochondrial membrane potential. Cell apoptosis was further confirmed by the release of cytochrome c, the up-regulation of pro-apoptotic proteins (BAX), the downregulation of anti-apoptotic protein (BCL) and the activation of caspase-3 and -9 [27].

### 3.2. Flavone

The main structure of flavones consists of a double bond between carbons 2 and 3 and a carbonyl group in position 4 of the C ring. Flavones are most present in fruit peels, celery, parsley, chamomile, tomato peel and honey [76]. These types of flavonoids are usually pale yellow in colour, and the most popular forms are apigenin and chrysin, especially in high-grade adult-type diffuse glioma studies [77]. Other flavone examples include luteolin and diosmin.

#### 3.2.1. Chrysin

Chrysin, which is also known as 5,7-dihydroxyflavone, is found abundantly in blue passion flower (*Passiflora*), honey, propolis and several medicinal plants and fruits, such as *Diaphragma juglandis fructus*, *Hphaene thebaica*, *Chaetomium globosum* and *Cytisus villosus*, bitter melon (*Momordica charantia*) and wild Himalayan pear (*Pyrus pashia*) [48]. Chrysin’s chemical structure consists of hydroxyl groups attached to the A aromatic ring at carbons 5 and 7.

In a study by Chia-Leng et al., chrysin sensitizes GBM8901 cells to TMZ by inhibiting autophagy [78]. Chrysin markedly inhibited TMZ-induced LC3-II formation, Atg7, Atg12-Atg5 conjugate, beclin-1, and p62, thereby inhibiting autophagy. Other than that, chrysin has been shown to suppress MGMT expression by inhibiting AKT protein expression in a dose-dependent manner [78]. Chrysin is able to induce apoptosis in U251 cells by increasing p53 protein expression levels and PARP cleavage. The cell cycle distribution also showed an increase in the SubG0 fraction, with an increase in the percentage of cells in early and late apoptosis as well as necrosis. A reduction in antiapoptotic proteins such as Bcl-2 and Bcl-xL and the upregulation of proapoptotic proteins such as Bax, Bad and Bak after chrysin treatment were also observed in other studies [28]. Chrysin treatment can also suppress HIF1α, subsequently inhibit expression of PDK1, PDK3, and GLUT1, and promote cell death under both hypoxia and normoxia [79]. A combination of chrysin and TMZ led to a double reduction of NF-κB nuclear localization (p65 and p50) in U87-MG cells [80].

Studies also reported the anti-migration effect of chrysin by regulating MMP-2 expression in GL-15 cells [81]. Other than that, chrysin promoted TMZ treatment efficacy and inhibited C6, T98, U251, U87 and A-172 cell proliferation by activating p38 MAPK and downregulating the protein expression of p-Erk1/2 [28,32,82]. Chrysin also inhibited the proliferation, migration and invasion capacity of U87 cells by suppressing the translocation of nuclear factor erythroid 2 (NF-E2)-related factor 2 (Nrf2) into the nucleus [32]. The inhibition of Nrf2 can further suppress the expression of hemeoxygenase-1 (HO-1) and NAD(P)H quinine oxidoreductase-1.

#### 3.2.2. Apigenin

Apigenin (4′,5′,7-trihydroxyflavone) is a naturally occurring plant flavone with molecular structure C15H10O5 [83]. Apigenin is abundantly present in chamomile tea (*Matricaria chamomilla*) and other common fruits, plant-derived beverages, and vegetables. Apigenin’s chemical structure is similar to chrysin, except it consists of hydroxyl groups attached to the B aromatic ring at carbons 4.

Apigenin has been shown to inhibit high-grade adult-type diffuse glioma cell proliferation and survival by suppressing the expression of Erk in U87-MG and U1242MG cells [24]. Apigenin inhibited high-grade adult-type diffuse glioma cell proliferation and survival but did not have any effects on normal human astrocyte cells. Data from other studies also supported the evidence that apigenin induced T98G and U87MG cell apoptosis with increased phosphorylation of p38 MAPK and the activation of the JNK1 pathway [84]. The combination of apigenin with TMZ treatment significantly induced G2 phase cell cycle arrest by inhibiting protein expression of p-AKT, cyclin D1, Bcl-2, MMP-2 and MMP-9 [85]. Downregulation of p-AKT and MAPK by apigenin treatment further reduces the expression of CD133, Nanog and Sox2 [86].

Apigenin regulates the autophagy activities by increasing the LC3II/I ratio and expressions of Atg-7, Atg-5 and beclin-1 levels and decreasing p62 levels in U87-MG cells [87]. Apigenin also enhanced PARP cleavage at 40 and 80 μM, with the indication of loss of DNA repair and G2/M cell cycle arrest [24]. In the same study, apigenin increased the activation of caspase-3, -8 and -9 to initiate both extrinsic and intrinsic apoptosis mechanisms in U87-MG cells. Treatment of T98 cells with apigenin increased pro-apoptotic Bax protein while decreasing anti-apoptotic Bcl-2 expression at mRNA and protein levels [84]. Apigenin acted as a casein kinase-2 (CK2) inhibitor and decreased MDM2-p53 association and p53 ubiquitination to enhance p53 levels. Subsequently, apigenin increased the expression of p53 target genes associated with cell cycle progression and apoptosis, such as p21, GADD45β and Noxa in A172 cells [33].

### 3.3. Flavanone

Flavanones, also called dihydroxyflavone, have the C ring saturated; therefore, there is no double bond between positions 2 and 3, and this is the only structural difference between flavanones and flavones [12]. Flavanones are an important class that is generally present in all citrus fruits such as oranges, lemons and grapes. Examples of flavanone include hesperidin, naringenin, and eriodyctiol, while naringenin and silibinin are the principal examples against high-grade adult-type diffuse gliomas.

#### 3.3.1. Naringenin

Naringenin is a white crystalline powder that lies under the flavanone group and is chemically called (2S)-5,7-dihydroxy-2-(4-hydroxyphenyl)-2,3-dihydrochromen-4-one [50]. Naringenin is predominantly found in edible fruits such as Citrus species, tomatoes, and figs belonging to the Smyrna-type Ficus carica. Naringenin has the skeleton structure of a flavanone with three hydroxyl groups at the 4′, 5 and 7 carbons, a carbonyl group on the C ring, and a 5-OH group on the A ring, which are responsible for the antioxidant activity of naringenin [29].

Naringenin was found to repress Erk1/2 and p38 activities at concentrations of 100, 200 and 300 μM. The inhibition of Erk and p38 was then found to be involved in MMP-2 and MMP-9 expression, which revealed naringenin’s ability to inhibit the migration of glioblastoma cells [88,89,90,91]. Decreased levels of MMP-2 and -9 were found to correlate with increased expression of TIMP-1 and -2. A single treatment of naringenin increased the upregulation of caspase-8 and caspase-3 and the downregulation of Bcl-2 in C6, U87-MG and LN228 cell lines. When naringenin was combined with TMZ, there was a greater increase in the levels of caspase-8 and caspase-3, while the levels of Bcl-2 were downregulated [90,91]. Other than that, naringenin also decreased the expression of Gli-1 and Smo and upregulated the expression of Sufu to prevent proliferation and metastasis in C6 cells [92].

#### 3.3.2. Silibinin

Silibinin is also called silybin, exists in two diastereomers (silibinin A and silibinin B) and is chemically composed of two main units, which are linked to one structure by an oxeran ring [93]. The first unit is a taxifolin unit (a flavonoid unit), while the second is a phenylpropanoid unit (a conyferil alcohol), and these determine its behaviors. Silibinin is mostly derived from the milk thistle plant (silybum marianum) [51].

Silibinin was shown to inhibit GBM cell migration by inhibiting MMP-2 and -9 [34,94]. The combination of silibinin and ATO inhibited U87-MG cell migration not only through the downregulation of MMP-2, MMP-9, MT1-MMP, and bcl-2 and the upregulation of caspase-3 [95]. Silibinin treatment also promoted A172 cell apoptosis through the cleavage of PARP-1 and caspase 3 in concentration and time-dependent manners [96]. Furthermore, silibinin treatment in U87-MG and T98G cells was found to be correlated with the downregulation of iNOS [97]. The downregulation of iNOS led to the upregulation of caspase-8 and p53 protein, resulting in the activation of a caspase cascade via Bid cleavage, leading to the induction of apoptosis. In addition to that, treatment with silibinin has been shown to downregulate the expression of LC3BI, LC3BII and beclin-1, which in turn suppressed autophagic activity in U87-MG and T98G cells [34].

### 3.4. Flavanol

Flavanols, also called dihyroflavonols or catechins, are the 3-hydroxy derivatives of flavanones [12]. They are also referred to flavan-3-ols, as the hydroxyl group is always bound to position 3 of the C rings. There are eight catechins: C ((-)-catechin), EC ((-)-epicatechin), ECG ((-)-epicatechingallate), EGC ((-)-epigallocatechin), EGCG ((-)-epigallocatechin gallate), GC ((-)-gallocatechin), CG ((-)-catechingallate), and GCG ((-)-gallocatechingallate) [98]. Flavanols are found abundantly in tea, cocoa and berries to which a potent antioxidant activity is ascribed, especially for epigallocatechin-3-gallate (EGCG), which is the most well-studied flavanol in high-grade adult-type diffuse glioma cells [99].

#### EGCG

Among all the catechins, EGCG is the most abundant and biologically active green tea catechin, which accounts for 50–80% of the total catechins in green tea [100]. As the structure of EGCG possesses a gallic acid, the presence of the gallate moiety and the extra phenol ring on EGCG enhances its anticancer property, which traps electrons as a scavenger of free radicals and reduces oxidative stress [101].

Treatment with EGCG has been shown to increase the phosphorylation of p38 MAPK and JNK1 in T98G and U87-MG cells [84]. A study also reported the ability of EGCG to suppress the mRNA and protein expression of MGMT, therefore reversing TMZ resistance in MGMT-positive GBM-XD and T98G cells. Meanwhile, EGCG enhanced MGMT expression in the nontumor glial cells through the inhibition of DNMT1 and demethylation of the MGMT promoter [102]. This finding showed that EGCG can preferentially inhibit MGMT in high-grade adult-type diffuse glioma cells rather than in nontumor glial cells. EGCG also induces U87-MG cell apoptosis through the downregulation of PARP cleavage and Bcl-2, upregulating Bax in a dose-dependent manner [103]. Other than that, EGCG can inhibit MT1-MMP and its interactors in the U87 cell line [104]. MT1-MMP is a transmembrane MMP that triggers intracellular signaling and regulates matrix proteolysis, subsequently regulating tumor-associated angiogenesis and inflammation. This finding is in line with other studies, which have found that ECGC downregulated T98G expression of MMP-2 and -9 in a dose-dependent manner [35,105,106,107].

EGCG activates caspase-8 and the cleavage of Bid to tBid in U87-MG and T98 cells [84]. Caspase-8 activation supports the Bax-mediated mitochondrial release of cytochrome C. Additionally, a significant increase in the expression of caspase-3 and LC3BII was observed in GBM cells treated with 50 and 100 μM EGCG [108,109]. Furthermore, a combination of TRAIL with EGCG was found to significantly increase active cleaved caspase-7 in both U87-MG and A172 cells [110]. ECGC has been found to significantly improve the therapeutic effect of TMZ by decreasing the expression levels of glucose-regulated protein 78 (GRP78) [36,111]. GRP78 is an ER stress marker that regulates the balance between proliferation and apoptosis by sustaining ER protein folding capacity and maintaining ER- stress sensors and associated pro-apoptotic proteins in their inactive state. Moreover, there were studies that reported potential EGCG as an inhibitor for the tyrosine phosphorylation of PDGF-Rbeta and its downstream signaling pathway in A172 cells with a dosage of 20–50 microM [112,113].

### 3.5. Isoflavone

Isoflavones are a distinctive but large subgroup of flavonoids with structural similarities to estrogens15 and to 17-β-estradiol [114]. Isoflavones are soy phytoestrogens and biologically active components of several agriculturally important legumes such as soy, peanut, green peas, chickpeas and alfalfa. Some isoflavones were reported to be present in microbes and play an important role as precursors for the development of phytoalexins during plant-microbe interactions [115]. Subclasses of isoflavone include daidzein, genistein, glycitein and biochanin A. Among all the isoflavones, genistein and biochanin A are the most well-studied compounds, which can be ascribed to their influences in various high-grade adult-type diffuse glioma pathways.

#### 3.5.1. Genistein

Genistein is chemically known as 5,7-dihydroxy-3-(4-hydroxyphenyl)chromen-4-one, with a molecular formula of C15H10O5 and a molecular weight of 270.241 g/mol. The basic carbon skeleton of genistein has a double bond between positions two and three. Additionally, it has an oxo group at position four of ring C and three additional hydroxyl groups at positions five and seven of ring A and position four of ring B. The best-known sources of genistein are soy-based food, such as tofu, soybeans, kudzu and lupin [53].

In Regenbrecht‘s study, the p53 signaling pathway is one of the top five most enriched pathways induced by genistein. Followed by the downregulation of the p53 pathway, the expression pattern of cyclin A and B were downregulated with both CDK1 and CDK2, which inhibited the transition through the G1 phase of the cell-cycle [116]. Moreover, genistein was found to be a specific inhibitor to disrupt ZDHHC17-MAP2K4 complex formation for T98G and U87MG cell proliferation and GSC self-renewal. MAP2K4 is responsible for the activation of JNK and p38 [84,117]. Genistein can also bind to DNA-PKcs and inhibit the DNA-PKcs/Akt2/Rac1 signaling pathway to reduce GBM cell invasion and migration both in vitro and in vivo [118]. The inhibition of DNA-PKcs phosphorylation activities further deactivated the non-homologous end joining (NHEJ) and homologous recombination (HR) repair mechanisms, consequently leading to apoptosis [119]. Genistein treatment also induced growth arrest at the G2/M phase in association with telomerase inhibition via the inhibition of TR- and TERT mRNA [37].

#### 3.5.2. Biochanin A

Biochanin A, chemically known as 4′-methoxy-5, 7-dihydroxy isoflavone, is the most abundant isoflavone with the highest yield of 15.7 mg/150 mg among other isoflavones [54]. The major sources of biochanin A include *Cicer arietinum*, *Trifolium pratense*, *Casia fistula*, *Dalbergia paniculate*, *Arachis hypogaea*, *Medicago sativa*, and *Astragalus membranaceus* [54].

Biochanin A was shown to impair autophagy, as evidenced by a decrease in LC3 puncta and the conversion of LC3-I to LC3-II in U251 cells. In the same study, there was a decrease in beclin-1 and an increase in p62 after biochanin A treatment for 48 h. Furthermore, the combination of biochanin A with TMZ induced a higher ratio of apoptotic cell death through the downregulation of LC3 levels [120]. A study has shown that biochanin A sensitized U251 and U87 cells to TMZ by enhancing the expression of Bax and suppressing the expression of Bcl-2 to induce apoptosis [120]. Other than Bax and Bcl-2, biochanin A also augmented the effect of TMZ by increasing the expression of p53, decreasing the levels of p-Erk and c-myc, thereby inhibiting U87MG and T98G cell proliferation and growth [38]. Furthermore, biochanin A was shown to enhance the inhibitory effect of TMZ and genistein on MT1-MMP, MMP-9, MMP-2 and uPAR, which then inhibited U87-MG cell migration [38,121]. Biochanin A also sensitized the cytotoxicity of rapamycin by decreasing the phosphorylation of Akt and eIF4E proteins in U87 cells [122].

### 3.6. Anthocyanin

Anthocyanin is a subclass of flavonoids that is in the glycoside form of anthocyanidins [123]. The basic chemical structure of anthocyanins is a flavylium cation that links hydroxyl and methoxyl groups with one or more sugars [123]. The conjugated bonds of anthocyanins resulted in blue, red, or purple pigments responsible for colors in plants, flowers and fruits [12,123,124]. They occur predominantly in the outer cell layers of various fruits such as cranberries, black currants, red grapes and blackberries. Aside from being traditionally used as dye and food colorants, they are strong antioxidants and potential functional foods that have been consumed to prevent and treat various diseases [124]. Anthocyanin possesses antidiabetic, anti-inflammatory, antimicrobial as well as anticancer effects. Cyanidin-3-O-glucoside (C3G) is the major anthocyanin found in legumes, and it has potential as an anti-cancer agent.

#### Cyanidin-3-O-Glucoside

Treatment of C3G resulted in 32% apoptotic U87 cells after 24 h. Apoptosis is induced by increasing p53 and Bax and decreasing Bcl-2 levels [125]. Other than that, C3G upregulated miR-214-50 and enhanced the cytotoxic effect of TMZ in the LN18/TR glioma cell line. MiR-214-50 mimics downregulated MGMT expression, thereby decreasing glioma cell invasion and migration [126]. Other mechanistic studies also revealed that C3G can suppress the neuroinflammatory response via JNK activation [127].

## 4. Interaction Analysis of Common Molecular Targets of Flavonoids

**Figure 2:**
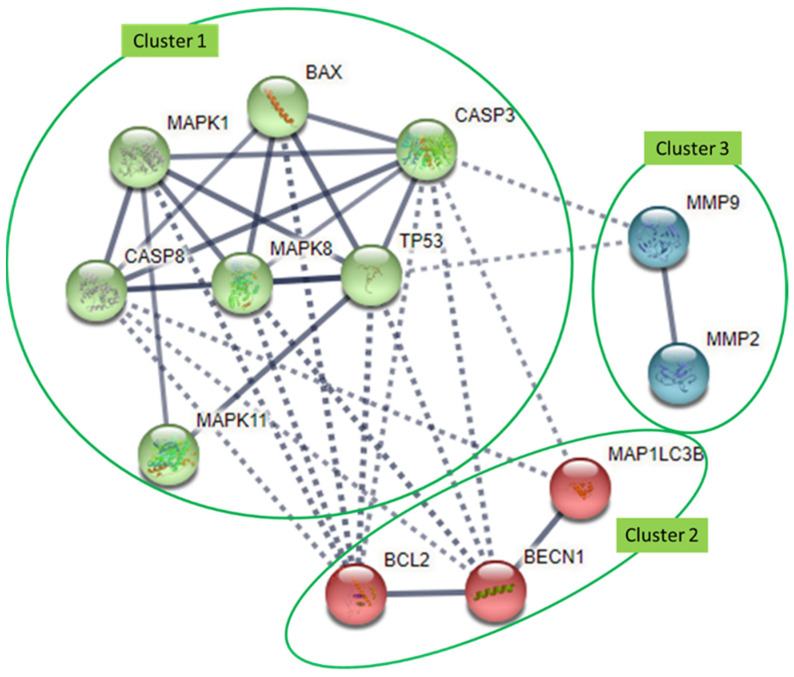
Protein-protein interaction (PPI) networks of molecules targeted by at least 5 flavonoid compounds were generated by STRING software (15 nodes, 47 edges). The nodes represent proteins, and the protein structures are visible in the nodes. The lines connecting the nodes indicate the association between the proteins (solid lines: association of proteins between same cluster; dotted lines: association of proteins between different clusters). The thicker the line, the higher the degree of confidence prediction of the interaction. K-means clustering tool in this software was used to group molecules into three distinct clusters. Those shown in green (cluster 1) are related to cell proliferation, differentiation and apoptosis, those shown in red (cluster 2) are involved in cell apoptosis and autophagy, and those shown in blue (cluster 3) are related to cell migration and invasion.

Overall, as discussed earlier, a sum of 50 molecular targets were modulated by the flavonoid compounds and are summarized in Table 2. Among the 50 molecular targets, there were 12 molecules commonly targeted by at least 5 out of a total 10 flavonoid compounds (Table 2). These 12 molecular targets include LC3B, beclin-1, MMP-2, MMP-9, p53, Bcl-2, Bax, caspase-3, caspase-8, JNK, Erk and p38. Interactions between these 12 targets were analyzed using the STRING database. A total of 12 nodes and 33 edges were embodied with an average node degree of 5.5 and an average local clustering coefficient of 0.786, which is shown in Figure 2. The k-means clustering tool was used to group these molecular targets into three different clusters (Figure 2). The molecular targets in cluster 1 are Bax, p38 (MAPK11), Erk (MAPK1), JNK (MAPK8), TP53 (p53), caspase-3 and caspase-8, which are involved in proliferation, differentiation and apoptosis, while cluster 2 consists of Bcl-2, beclin-1 and LC3B, which are involved in regulating apoptosis and autophagy. Cluster 3 contains only two molecular targets, which are MMP-2 and MMP-9, which are responsible for cell migration and invasion.

All these molecules have a strong interaction with each other in their own cluster. For instance, apoptotic proteins such as caspase-8, caspase-3, Bax and p53 were shown to strongly interact with p38, Erk and JNK in the MAPK cascade, which regulates cell proliferation, survival and differentiation in cluster 1. In cluster 2, the anti-apoptotic Bcl-2 protein shows a strong connection with the autophagy-related protein beclin-1, while beclin-1 strongly interacts with LC3B. In cluster 3, MMP-2 and MMP-9, which are responsible for cell migration and invasion, showed strong interactions with each other. As shown in Figure 2, molecules between clusters 1 and 2 still exhibit interactions, while MMPs in cluster 3 only interact with caspase-3 and TP53 in cluster 1. Caspase-3 in cluster 1 showed the highest interaction with other molecules, including Bax, MAPK1, caspase-8, MAPK8, and TP53, as well as Bcl-2, beclin-1 and LC3B in cluster 2 and MMP-9 in cluster 3. MMP-2 showed the least interaction among all the molecules, as it only interacted with MMP-9.

**Table 2:** The molecular targets modulated by flavonoid subclasses: flavones, flavonols, flavanols, flavanones, isoflavones, and anthocyanidins and their compounds: quercetin, rutin, chrysin, apigenin, naringenin, stilbene, ECGC, genistein, biochanin A and C3G in high-grade adult-type diffuse glioma. * represents common targets of flavonoid compounds. ↑ represents upregulation; ↓ represents downregulation of molecular targets.

Cellular Activities	Molecular Targets	Flavonols		Flavones		Flavanones		Flavanols	Isoflavones		Anthocyanidins	Glioma Model	References
		Quercetin	Rutin	Chrysin	Apigenin	Naringenin	Silibinin	EGCG	Genistein	Biochanin A	C3G	(In Vitro or In Vivo)	
Autophagy	Atg7	↓		↓	↑							U251, U87, GBM8901	[57,78]
	LC3B *	↓	↓	↓	↑		↓	↑		↓		U373MG, T98, D54-MG, U87-MG, U251-MG, GBM8901	[30,31,65,87]
	Beclin-1 *	↓		↓	↑		↓			↓		U373MG, T98, T98G, GBM8901	[30,65,66,78]
	p62			↓	↓					↓		GBM8901	[78]
DNA repair	MGMT			↓				↓			↓	GBM8901, T98G, U87-MG, LN18/TR	[78,84,102,126]
	PARP	↓		↓	↓			↓				T98G, U373, C6, U251	[28,30,48,65]
Cell migration, invasion, adhesion	uPAR	↓		↓	↓					↓		U87-MG	[38,121]
	MMP-2 *				↓	↓	↓	↓		↓		GL-15, T98G, U87MG	[81,85]
	MMP-9 *	↓			↓	↓	↓	↓		↓		U87, T98G, U87MG	[58,59,60,85]
Cell apoptosis	p53 *	↑	↑	↑	↑		↑		↑	↑	↑	T98G, U373, C6, U-118, LN-229, U251	[27,30,48]
	Bcl-2 *		↓	↓	↓	↓	↓	↓		↓	↓	A-172, T98G, U87MG	[28,84,85,90,91]
	Bcl-xL		↓	↓								U-118, LN-229, A-172	[27]
	Bax *		↑	↑	↑					↑	↑	U-118, LN-229, A-172	[27,28,84,108,109]
	Bad			↑								A-172	[28]
	Bak			↑								A-172	[28]]
	p65			↓								U87-MG	[80]
	p50			↓								U87-MG	[80]
	Caspsase-3 *	↑	↑		↑	↑	↑	↑				T98G, U373MG, U-118, LN-229	[26,30,35,65,67,68,69]
	Caspase-7							↑				U87-MG. A-172	[110]
	Caspase-8 *				↑	↑	↑	↑	↑			C6, U87-MG, LN228	[90,91]
	Caspase-9	↑	↑		↑							T98G, U373, C6, U-118, LN-229	[27,35,69]
	Rb	↓										U87MG, U373MG, T98G,	[23,70]
	Cyclin A								↓				
Cell cycle arrest	Cyclin D	↓			↓		↓		↓			T98G, U87MG	[85]
	CDK1								↓			U373, GBM1207	[116]
	CDK2								↓			U373, GBM1207	[116]
Oxidative stress	Nrf2			↓								U87	[32]
	HIF-1			↓								U251, GBM28, GBM37	[79]
Cell proliferation, survival, differentiation, apoptosis	PDK1			↓								U251, GBM28, GBM37	[79]
	PDK3			↓								U251, GBM28, GBM37	[79]
	Raf	↓										DBTRG-05, U251	[62,63]
	JNK *	↑			↑							U87-MG, D54-MG, U251-MG, T98G, U87MG	[30,74,84,127]
	Akt	↓		↓	↓							U87, GBM8901, T98G, U87MG	[58,63,78,85]
	Erk *	↓		↓	↓	↓		↑	↓		↓	U87, U87-MG, U251, DBTRG-05MG, GL-15, C6, T98, A-172, U1242MG	[24,28,32,58,74,75,82]
	CK2				↓					↓			
	p38 *	↓		↑	↑	↓				↓		C6, T98, U251, U87, A-172, T98G, U87MG	[28,32,62,63,82,84]
	GLUT1			↓				↑	↓			U251, GBM28, GBM37	[79]
	IL-6	↓										U251, TG1	[64]
	IL-10	↓										U251, TG1	[64
	STAT3	↓										U87MG, U373MG, T98G	[23,70]
	GRP78							↓				U373Mg, U87MG	[36,111]
	PDGF-Rbeta							↓				A-172	[112,113]
	Gli-1					↓						C6	[92]
	Smo					↓						C6	[92]

## 5. Clinical Relevance of Flavonoid Molecular Targets in High-Grade Adult-Type Diffuse Glioma

**Figure 3:**
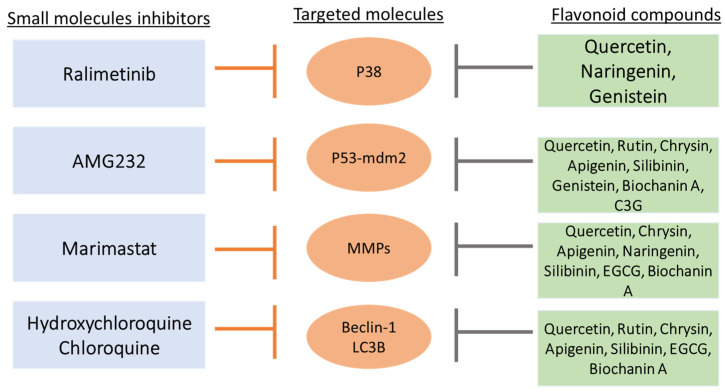
Common molecular targets of small molecule inhibitors and flavonoid compounds.

To date, no clinical trial has been conducted to evaluate the efficacy of flavonoids on high-grade adult-type diffuse glioma patients. However, there are several small molecule inhibitors used in clinical trials that also target similar and common molecular targets as flavonoids (Figure 2 and Figure 3) [128,129,130,131,132]. These small molecule inhibitors are ralimetinib (a p38-MAPK inhibitor), AMG232 (an mdm2-p53 inhibitor), marimastat (MT) (an MMP inhibitor), hydroxychloroquine (HCQ) and chloroquine (CQ) (beclin-1 and LC3B autophagy protein inhibitors) [128].

Ralimetinib inhibited the expression of p38-MAPK (in cluster 1) in high-grade adult-type diffuse glioma patients, and this molecular target was also inhibited by flavonoid compounds such as quercetin, naringenin and genistein (Figure 3) [84,88,89]. The MAPK superfamily comprises Erk, JNK and p38, which are responsible for basic cellular processes, including cell proliferation and differentiation [133]. Among the MAPK superfamily, the p38-associated pathway-MAPK is highly altered and associated with therapy resistance that confers poor survival in high-grade adult-type diffuse glioma [134,135,136,137,138,139]. The phosphorylation level of MAPK can be used to predict the prognostic and survival rate of patients with GBM treated with TMZ [134]. Ralimetinib has been used in combination with radiotherapy and chemotherapy (TMZ) in the treatment of newly diagnosed GBM patients [129]. In this phase I clinical trial, the maximum tolerated dose (MTD) of ralimetinib (p38 inhibitor) was 100 mg/12 h, while at the MTD, toxicities of grade ≥3, including hepatic cytolysis, dermatitis/rash, lymphopenia and nausea, was observed with limited activity in GBM patients. Only around 54% reduction of pMAPKAP-K2 was observed in patients’ peripheral blood mononuclear cells.

Moreover, AMG232 inhibited the binding of MDM2 to TP53 protein (in cluster 1), which also can be targeted by quercetin, rutin, chrysin, apigenin, silibinin, genistein, biochanin A and C3G in in vivo studies (Figure 3) [33,34,116,125]. TP53 encodes for the p53 protein, which is a tumor suppressor protein that induces cell apoptosis to prevent damaged cells from further proliferating. P53 is activated upon DNA damage to increase the expression of pro-survival proteins such as Bcl-2 and inhibit the expression of the pro-apoptotic Bax protein [140,141]. The inhibition of Bcl-2 or the upregulation of Bax can further activate the cleavage of caspase-3 and caspase-8, which then induces apoptosis. MDM2, a negative regulator of p53, binds to p53 and leads to p53 ubiquitination and proteasomal degradation. According to TCGA, the p53-mdm2 pathway is one of the most commonly dysregulated pathways in 84% of GBM patients [142,143]. GBM patients with mutated p53 were shown to have lower overall survival compared to wild-type p53 patients [144]. Clinical studies also demonstrated that knockdown of the mutant p53 can lead to a 5-fold increase in chemosensitivity to TMZ. In one of the recent clinical studies, AMG232 stabilizes the progression of GBM in patients with p53 wild type, with an overall median duration of stable diseases of 1.8 months [145]. However, dose-limiting toxicities such as thrombocytopenia and neutropenia are still detected in patients.

Furthermore, HCQ and CQ are autophagy inhibitors that can modulate autophagy-related proteins such as beclin-1 and LC3B, which are categorized in cluster 2 (Figure 2). Beclin-1 and LC3B were also commonly targeted by quercetin, rutin, chrysin, apigenin, silibinin, EGCG and biochanin A in pre-clinical studies [30,57,109]. LC3B and beclin-1 are autophagy-related proteins that play an important role in regulating autophagic activities. Beclin-1 acts during the initiation stage of autophagy by forming the isolation membrane to form the autophagosome, a double-membrane structure that degrades cytoplasmic material. The free form of LC3-I is converted to the LC3-II form after being cleaved by autophagin, which is activated by ROS, leading to autophagy. Evidence from high-grade adult-type diffuse glioma models demonstrated that autophagy is involved in tumor progression and can affect the response to treatment [146]. In a pre-clinical study, HCQ at less than 5 μg/mL led to the accumulation of LC3B-II proteins and inhibited the autophagy process, which was found to potentiate the anti-cancer effect of bevacizumab in GBM cells [147]. HCQ has also been shown to decrease the formation of autophagosome bodies by reducing beclin-1 expression [128]. CQ has also been found to augment the anti-glioma effect of TMZ monotherapy by inhibiting the conversion of LC3-I to LC3-II in GBM cells [148]. Pre-clinical evidence has shown that GBM patients may benefit from combining HCQ or CQ with conventional therapy, which inhibits autophagy, thereby sensitizing alkylating drugs’ cytotoxic effects [149,150]. However, several clinical trials have disproved the use of HCQ or CQ with TMZ due to the significant number of adverse events such as blurred vision, ECG QT-corrected interval prolongation, retinal toxicity, and cardiac conduction disturbances [130,131]. In one of the clinical trials, the MTD of CQ in GBM patients was 200 mg in combination with chemoradiation, with a median overall survival rate of only 16 months [131]. HCQ was preferred over CQ as the maximum tolerated dose of HCQ was 600 mg/d in combination with radiation therapy and TMZ in GBM patients after initial resection [130]. At 600 mg/d, although the pharmacokinetic analysis showed a significant increase in autophagic vacuoles and LC3B in the peripheral blood mononuclear cells, it was not consistent in all patients, and no significant improvement in overall survival was observed.

Marimastat targeted and inhibited multiple MMPs that were grouped in cluster 3 (Figure 2). Flavonoids such as quercetin, chrysin, apigenin, naringenin, silibinin, EGCG and biochanin A have also been shown to target MMPs in high-grade adult-type diffuse glioma cells in pre-clinical studies [58,81,85,88,95,122]. MMPs are metalloproteinases that are calcium-dependent zinc-containing endopeptidases that can digest various extracellular matrix (ECM) macromolecules that play a vital role in high-grade adult-type diffuse glioma invasion [151]. In several pre-clinical and clinical studies, MMPs are shown to upregulate malignant gliomas, and the level of MMPs is correlated with malignant progression [152,153,154]. Higher expression of MMPs was associated with shorter overall survival in patients with grade III and IV astrocytic tumors (HR 1.6) [155,156]. MT, which is a broad-spectrum matrix inhibitor of several MMPs, including MMP-2 and MMP-9, has been used in one clinical trial. However, the study outcomes were not positive, as MT did not improve survival in patients with glioblastoma following surgery and radiotherapy [132]. The median survival time for the placebo group was 37.9 weeks and 42.9 weeks for the MT group, with an HR of 1.16. No statistically significant differences were found between these groups with respect to survival time [132]. Moreover, musculoskeletal toxicities led to dose modifications or withdrawal in 20% of MT-treated patients [132].

Although a few clinical studies have been conducted on small molecule inhibitors against high-grade adult-type diffuse glioma, the results were unfavorable mostly due to dose-limiting toxicities or even the exacerbation of chemo-related hematological toxicity, which inhibits their translation into clinical practice [145,157]. To overcome these challenges, natural compounds such as flavonoids have been gaining immense attention and have been widely studied pre-clinically. In pre-clinical studies, quercetin targeted all of the mentioned molecules (p38, p53, MMPs, beclin-1 and LC3B) that were found to be monotargeted by ralimetinib, AMG232, MT, HCQ and CQ. Besides quercetin, other compounds such as chrysin, apigenin, silibinin and biochanin A were also shown to inhibit most of the molecules (p53, MMPs, beclin-1 and LC3B), except p38, regarding high-grade adult-type diffuse glioma cell proliferation, migration and differentiation. Naringenin, genistein, rutin, and EGCG also demonstrated similar effects with at least one of the listed small molecular inhibitors: ralimetinib, AMG232, MT and HCQ. Overall, all of the listed flavonoids in Table 1 demonstrated the same anti-high-grade adult-type diffuse glioma effects with the small molecular inhibitors (Figure 3). It is worth noting that by modulating p38, p53, MMPs, beclin-1 and LC3B activities, the expression of other proteins such as Erk, JUN, caspase-3, caspase-8, Bax and Bcl-2 would also be affected (Figure 2). Moreover, flavonoids, unlike small molecule inhibitors, have been shown to exert preferential cytotoxicity against cancer over non-cancer cell lines [23,24,158]. Thus, introducing flavonoids into part of the current high-grade adult-type diffuse glioma treatments could potentially decrease the development of drug resistance and increase therapeutic efficacy.

## 6. Issues of Flavonoid Bioavailability and Methods to Overcome Them

Despite their promising ability to target different molecules involved in high-grade adult-type diffuse glioma cells, it is a known fact that flavonoids have limited bioavailability [39]. Several studies have reported that very low flavonoid concentrations were detected in blood, tumors, or extraintestinal tissues [159]. The low bioavailability of flavonoids has slowed down their translation into clinical use. Factors that affect the bioavailability of flavonoids include molecular weight, glycosylation, metabolic conversion and their interaction with colonic microflora [39]. Among all the common flavonoid compounds mentioned in this review, rutin is the largest compound and exhibits the highest molecular weight (610.517 g/mol). Because of its large molecular weight and size, the bioavailability of rutin was only ~20% of quercetin on the basis of area under the plasma concentration-time curve values and relative urinary excretions [160,161,162]. Moreover, the associated sugar moiety of flavonoids has a great influence on the absorption rate; it was reported that quercetin glucosides were absorbed 10 times faster and the plasma concentration peaked 20 times higher than quercetin rutinosides in humans [161]. It was suggested that glucosides can be absorbed in the small intestine instead of the colon after deglycosylation. Most flavonoids undergo metabolic conversions such as sulfation, methylation and glucuronidation in the enterocytes and liver, which results in low bioavailability. Furthermore, it was reported that most flavonoids were not absorbed in the small intestine. They reached the large intestine and were catabolized by colonic microflora into chain fission products that were mostly excreted into the feces [162,163]. In order to address these limitations, researchers have been making efforts to improve flavonoids’ bioavailability by introducing microencapsulation, nano-delivery systems, microemulsions, and enzymatic methylation techniques.

Among all, nano-drug delivery systems are the most widely used delivery technology, and the key goal is to efficiently deliver flavonoids to the intended pathological sites with minimal side effects. Manlio et al. synthesized a nanocarrier (nanohydrogel) of quercetin combined with TMZ to increase the internalization and cytotoxicity of quercetin in GBM cells [164]. This nanohydrogel, when loaded with quercetin, promoted preferential CD44 uptake by an active mechanism of internalization, thereby managing the A-172 and T98MG microenvironment and enhancing the therapeutic effect of TMZ. Quercetin delivered from a nanocarrier has shown significantly higher anti-inflammatory properties compared to unformulated quercetin, with abilities to reduce 72, 52, and 63.7% of IL-8, IL-6 and VEGF cellular secretion, respectively [164]. Gang Wang et al. demonstrated that PEG2000-DPSE-coated quercetin nanoparticles remarkably enhanced programmed cell death on C6 glioma cells through ROS accumulation and p53, cytochrome C and caspase-3 protein upregulation [165,166]. Their subsequent experiment revealed that PEG2000-DSPE polymeric liposomes loaded with quercetin and TMZ were rapidly taken up by U87 glioma cells compared to the free drugs [167]. An in vivo study also reported a significant accumulation of nanoliposomes containing quercetin and TMZ in the brain, with an increased plasma concentration of quercetin and TMZ as well as delayed clearance in a rat model of glioma. By increasing the biodistribution and prolonging the circulation times, quercetin and TMZ exerted enhanced antitumor effects by killing both drug-sensitive and drug-resistant U87 glioma cells. There were also other studies that reported the anti-glioma effects were improved when quercetin was loaded with nanoparticles [168,169,170,171,172,173,174,175].

The combination of paclitaxel and naringenin demonstrated more than 70% drug release and a significant improvement in drug absorption over the free drug suspension when loaded with solid lipid nanoparticles [176]. These drugs’ loaded-solid lipid nanoparticles also showed higher cytotoxicity on U87MG glioma cells compared to the free drug suspension. Maryam et al. demonstrated that a nanoformulation of silibinin with chitosan significantly inhibited C6 cell proliferation by increasing the expression of apoptotic genes (Bax and caspase-3) in comparison to free silibinin [177]. In this experiment, silibinin-coated chitosan nanoparticles exhibited a higher rate of release of silibinin in C6 cells (usually acidic) compared to H92C cells, as chitosan is insoluble in water but soluble in acidic conditions [177]. This controlled drug release characteristic with higher selectivity towards glioma cells over normal cells supports the promising use of these chitosan nanoparticles as a silibinin delivery system. Moreover, the bioavailability of genistein in a β-cyclodextrin formulation was significantly higher by up to 180% compared to free genistein in Sprague-Dawley rats [178]. Based on the results of the pre-clinical studies, the use of nano-drug delivery systems could certainly be clinically developed to improve flavonoids’ bioavailability and benefit high-grade adult-type diffuse glioma patients.

## 7. Conclusions and Future Perspective

Flavonoids naturally occurring in our daily diet are potential anti-high-grade adult-type diffuse glioma agents targeting different molecules. As multitargeted compounds, they could potentially break down the most challenging barrier—chemoresistance in the successful treatment of high-grade adult-type diffuse gliomas. Flavonoids’ targeted and regulated autophagy-related proteins, such as beclin-1 and L3CB, may be useful in reducing chemoresistance in GBM, especially TMZ-acquired resistance through cryoprotective autophagy. Moreover, flavonoids inhibited DNA repair mechanisms such as MGMT and PARP, which were found to be overexpressed in most chemoresistant high-grade adult-type diffuse glioma cells. It is worth noting that flavonoids, unlike the small molecule inhibitors, can target most of the molecules (including caspase-3, which was found to be the most interactive protein from the PPI networks) involved in high-grade adult-type diffuse glioma with minimal side effects. Besides being a single treatment in high-grade adult-type diffuse glioma, the combination of flavonoids (apigenin, quercetin, chrysin, rutin, naringenin, EGCG and biochanin A) with TMZ was able to induce a higher cytotoxic effect, as reported in a few pre-clinical studies [74,78,90,120].

Among all the flavonoid compounds mentioned earlier, quercetin was shown to modulate most of the molecules mentioned earlier, including the most interactive protein, caspase-3. By targeting those molecules, quercetin can regulate high-grade adult-type glioma cell autophagy, DNA repair mechanisms, cell motility, apoptosis, cell cycle arrest, proliferation and differentiation. More importantly, quercetin was able to modulate similar molecules (p38-MAPK, p53-mdm2, MMPs and autophagy proteins LC3B and beclin-1) as shared by the small molecule inhibitors. Based on our review, we would like to suggest quercetin as one of the main compounds to be focused on in future studies to facilitate its translation into clinical use. Other than being studied as a single treatment, few pre-clinical studies have reported that the combination of quercetin with conventional drugs such as TMZ enhanced the cytotoxic effect in high-grade adult-type diffuse glioma [26,67,68]. Numerous pre-clinical studies have also demonstrated that quercetin exhibits selective cytotoxicity in high-grade adult-type diffuse glioma cells against normal cells [179,180]. Several pre-clinical studies also reported that nanoparticles loaded with quercetin, when combined with TMZ, increase the cytotoxicity effects in both drug-sensitive and drug-resistant glioma cells [164]. However, the toxicity of quercetin (or other flavonoid compounds) and the efficacy of nanoparticle drug delivery systems in high-grade adult-type diffuse glioma patients still need to be evaluated in clinical trials. Taken together, further clinical studies on flavonoid treatment against high-grade adult-type diffuse glioma are required to provide a more comprehensive picture of flavonoids’ efficacy and toxicity in high-grade adult-type diffuse glioma patients.

## Figures and Tables

**Table 1:** Chemical structure of flavonoid compounds and their respective common source.

Flavonoid Subclasses	Examples	Common Source	References
Flavonol	(i) Quercetin		
	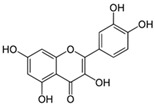	Oak (*Quercus*)	[46]
	(ii) Rutin (flavonol glycoside)		
	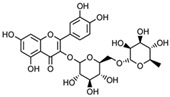	Rue (*Ruta graveolens*)	[47]
Flavone	(i) Chrysin		
	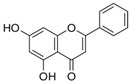	Passionflower (*Passiflora*)	[48]
	(ii) Apigenin		
	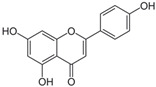	Chamomile tea (*Matricaria chamomilla*)	[49]
Flavanone	(i) Naringenin		
	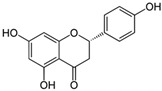	*Citrus* species	[50]
	(ii) Silibinin		
	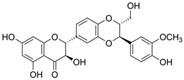	Milk thistle (*Silybum marianum)*	[51]
Flavanol	(i) EGCG		
	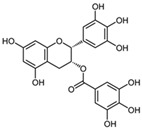	Green and white tea	[52]
Isoflavone	(i) Genistein		
	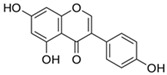	Soy-based foods	[53]
	(ii) Biochanin A		
	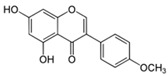	Chickpea (*Cicer arietinum*)	[54]
Anthocyanin	(i) Cyanidin 3-O-glucoside (C3G)		
	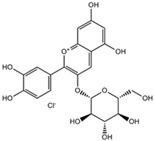	Red- and blue-pigmented fruits	[55]

## Data Availability

Data sharing not applicable. No new data were created or analyzed in this study. Data sharing is not applicable to this article.
